# Cross-cultural comparison of somatic-depressive symptom networks in Chinese and Rwandan adolescents: network analysis study

**DOI:** 10.1192/bjo.2026.12007

**Published:** 2026-06-26

**Authors:** Lisa Cynthia Niwenahisemo, Jian-yu Tan, Jin-hui Hu, Ming Ai, Xiao-ming Xu, Wo Wang, Patrick Remezo Mususa, Su Hong, Li Kuang

**Affiliations:** Department of Psychiatry, The First Affiliated Hospital of Chongqing Medical University, China; Department of Psychiatry, University-Town Hospital of Chongqing Medical University, China; Department of General Medicine, Université Catholique de Bukavu, The Democratic Republic of the Congo; Psychiatric Center, The First Affiliated Hospital of Chongqing Medical University, China

**Keywords:** Somatic symptoms, depression, adolescents, symptom networks, cross-cultural comparison

## Abstract

**Background:**

Adolescent depression often presents with somatic complaints, and its clinical manifestation is strongly shaped by cultural context. In non-western districts, psychological distress is frequently expressed through physical symptoms; a tendency that, combined with mental health stigma and culturally influenced health beliefs, complicates accurate detection, diagnosis and treatment. Standardised diagnostic tools developed in Western populations may overlook culturally specific symptom patterns, contributing to under-recognition and inadequate care. Despite the global impact of adolescent depression, cross-cultural symptom-level studies remain limited, hindering the development of culturally responsive mental health strategies.

**Aims:**

This study aims to compare somatic-depressive symptom networks in Chinese and Rwandan adolescents using symptom-level network analysis, to identify culturally distinct central and bridge symptoms, and to assess structural differences between symptom networks across groups.

**Method:**

A cross-sectional sample of 3830 adolescents (China: *n* = 2017, mean age 15.35 ± 1.56; Rwanda: *n* = 1813, mean age 15.80 ± 1.90) completed culturally adapted versions of the Patient Health Questionnaires for somatic symptoms (PHQ-15) and depression (PHQ-9). Gaussian Graphical Models were estimated in R to construct symptom networks. Centrality measures (expected influence and bridge expected influence) were used to identify influential symptoms within each group. Network Comparison Tests were conducted to examine differences in global strength and network structure, and bootstrapping was employed to assess network stability.

**Results:**

Depressive symptoms were more prevalent among Rwandan adolescents (54.6%) than among Chinese adolescents (29.2%), whereas somatic symptoms were more commonly reported by Chinese participants (71.0% *v*. 64.0%). Low energy and sleep problems emerged as key bridge symptoms in both groups. Cultural differences were observed in central symptoms: psychomotor impairment and chest pain were central symptoms in Rwanda, whereas dizziness and headaches were central in China. Network structure differed significantly between groups (*S* = 0.99, *p* < 0.05), with culturally specific symptom connections.

**Conclusions:**

The findings revealed distinct central and bridge symptoms in Chinese and Rwandan adolescents, reflecting culturally patterned architectures of symptom expression and distress reporting. These results highlight the need for culturally adapted screening tools and symptom-level interventions that target culture-specific symptoms to improve adolescent mental health care globally.

The underdiagnosis and misdiagnosis of adolescent depression represent a critical public health challenge, particularly in low-resource settings where mental health infrastructure is limited and early intervention remains underdeveloped.^1^ Depression is recognised as the leading global contributor to disability in young people, accounting for an estimated 49.4 million disability-adjusted life years (DALYs), and prevalent within 21% of adolescents worldwide.^2^ However, epidemiological data from non-Western regions may substantially underestimate the true burden, reflecting systemic gaps in detection and diagnosis rather than lower prevalence.

For instance, while epidemiological estimates suggest that 14–23% of Chinese adolescents experience depressive disorders,^3^ only approximately 4% are formally diagnosed in Rwanda. One contributing factor underlying this discrepancy is somatisation – the tendency for psychological distress to be expressed through physical symptoms. Across diverse cultural contexts, 70**–**90% of individuals with depression present primarily with somatic complaints such as fatigue, headaches, dizziness and sleep disturbances, which are strongly correlated with depression and anxiety severity but often mask underlying psychological distress.^4 **–** 9^ In Chinese out-patient settings, somatic symptoms are reported more frequently than psychological ones relative to Euro-Canadian populations, a pattern that persists across diagnostic revisions.^6,7^ Similar somatic-dominant presentations have been reported in several African countries,^9^ highlighting the transdiagnostic and transcultural relevance of somatisation. These findings underscore the need for more nuanced, culturally informed approaches to assessment, especially given the profound impact of adolescent depression on mental health, physical well-being and social development.^10^

Accurate diagnosis and effective treatment of depression are hindered when cultural variations in symptom presentation are not adequately considered.^11^ Cultural context shapes depressive symptomatology through at least two pathways, each contributing to diagnostic blind spots. First, through culturally shaped ‘idioms of distress’, such as dizziness and headaches among Chinese adolescents, which may reflect socialisation practices that limit emotional disclosure.^6,11^ Second, via culture-specific stressors that generate distinctive symptom clusters, as seen in Rwandan adolescents who frequently report chest pain and fatigue and often conceal psychological symptoms to avoid stigma.^12^ These presentations contrast with the cognitive-affective symptom profiles more commonly observed in Western populations,^13^ pointing to fundamental differences in how depression is experienced and communicated.

Applying modern diagnostic frameworks across cultures remains challenging, given that manifestations, interpretations and help-seeking behaviours are deeply embedded in local belief systems and social norms.^14^ This challenge is compounded by geographic disparities in research representation, with countries in Southern sub-Saharan Africa markedly underrepresented in cross-regional psychiatric comparisons.^15^ There is thus a pressing need for innovative, culturally sensitive methodologies to advance the global understanding of adolescent depression.

Network analysis offers a promising alternative to traditional latent variable models by conceptualising psychopathology as a dynamic system of interacting symptoms rather than as a reflection of underlying latent traits.^16,17^ Using contemporary model-selection techniques,^18^ researchers can estimate sparse, interpretable networks in which each node represents symptoms^19^ and edges reflect regularised partial correlations between them.^20^ This approach allows for the identification of central symptoms, those most influential in the network, and bridge symptoms that connect to different clusters of psychopathology. As such, network analysis provides not only a framework for investigating cultural differences in the structure of distress but also potential targets for culturally adapted intervention. Nevertheless, the symptom-level architecture of depression across cultures remains poorly understood, especially among adolescents.

To address these gaps, this study applies network analysis to compare somatic-depressive symptom networks in Chinese and Rwandan adolescents. Specifically, we aim to (a) identify central symptoms that drive overall network connectivity, (b) detect bridge symptoms linking depressive and somatic symptom communities and (c) compare network topology, edge weights and global strength across the two cultural groups. We hypothesise that Chinese and Rwandan adolescents will differ significantly in the prevalence and presentation of depressive and somatic symptoms,^21^ and that these differences will be reflected in culturally distinct patterns of symptom centrality and connectivity.

## Method

### Participants and sampling

This study employed a comparative design, collecting samples from secondary schools in the Shapingba District of Chongqing Municipality, China, and Kigali City, Rwanda. Both sites selected adolescent populations with typical cultural-psychological characteristics: the Chinese sample was drawn from an urban area with a high proportion of left-behind children, a group facing distinct familial stressors due to parental migration for work; the Rwandan sample was recruited from boarding schools in the capital city of Kigali, where students come from across the nation, representing a setting with notable mental health stigma and relatively limited service resources.

A stratified cluster sampling method was applied.^22^ In China’s S. District, 5 schools (3 public, 2 private) were first randomly selected from 28 secondary schools in the district, stratified by school type (public/private). Within each school, at least two classes were randomly chosen across grade levels, after which students were selected via computer-generated randomisation within classes, with attention to gender balance. In Kigali, Rwanda, 5 schools (2 public, 3 private) were randomly selected from 27 secondary schools in the city. In each school, several classes from grades 7 to 12 were randomly selected, and all students in those classes were surveyed. Sample size was estimated using G*Power software (version 3.1.9.7 for Windows 10; Heinrich-Heine-Universität Düsseldorf (Institute for Experimental Psychology), Düsseldorf, Germany; https://www.psychologie.hhu.de/arbeitsgruppen/allgemeine-psychologie-und-arbeitspsychologie/gpower) to ensure adequate power to detect medium effect sizes in cross-cultural network comparisons. A total of 4032 adolescents were recruited (China: *n* = 2120; Rwanda: *n* = 1912).

In the Chinese sample, questionnaires were completed during school-organised class sessions through a secure online platform (Chongyi Xinli). This platform supports anonymous submission and includes response logic checks to reduce invalid data. Each survey session was organised by two trained research assistants who provided standardised instructions, addressed technical questions and maintained proper conduct during the assessment.

In the Rwandan sample, paper questionnaires were administered in classroom settings, taking into consideration the local context of internet accessibility and electronic device availability. Prior to distribution, trained local researchers (all with backgrounds in psychology or public health) provided uniform explanations in Kinyarwanda. Students completed the questionnaires independently in class, and all forms were collected immediately afterward.

Both sets of questionnaires used locally adapted versions (Mandarin Chinese and Kinyarwanda) that had undergone translation and back-translation procedures.^23–25^ Before beginning the survey, all students received standardised instructions emphasising anonymity and voluntary participation. To ensure data quality, the following control measures were implemented at both sites: (a) setting completion time thresholds (responses completed in less than 10 min or more than 60 min were considered invalid); (b) including three attention-check items; (c) conducting a manual review of questionnaires with over 80% identical consecutive responses.

Recruitment was conducted at the class level, and all students present in the selected classes were invited to participate simultaneously. The number of students who declined participation or were absent was not systematically recorded; therefore, a conventional participation rate could not be calculated. All collected data were entered independently by two individuals, and a random check of 10% of the questionnaires was performed to verify consistency (agreement rate >99%). Ultimately, 103 invalid questionnaires from the Chinese sample (including incomplete responses, failed attention checks and patterned responses) and 99 from the Rwandan sample were excluded; among all students who began the survey, completion rates were high (95.1 and 94.8%, respectively).

This study complied with the ethical standards of the relevant national and institutional committees and adhered to the principles of the Declaration of Helsinki. Approval was obtained from the University of Rwanda Institutional Review Board (465/CMHS IRB/2022) and the Ethics Committee of Chongqing Medical University (2020-879). Both ethics boards gave specific attention to cultural differences in vulnerability assessment and confidentiality protection.

### Measures

Two well-validated self-report instruments were used. The Patient Health Questionnaire-9 (PHQ-9), a nine-item measure based on DSM-IV criteria, assessed depressive symptoms over the preceding 2 weeks using a four-point scale (0 = ‘Not at all’ to 3 = ‘Nearly every day’). Total scores range from 0 to 27, with established clinical cut-offs. Both the Chinese and Kinyarwanda versions have demonstrated strong psychometric properties in adolescent populations, including good internal consistency (α = 0.82**–**0.86) and criterion validity against clinical diagnoses.

Somatic symptoms were assessed using a culturally adapted 14-item version of the Patient Health Questionnaire-15 (PHQ-15), which excludes 1 sexually sensitive item. Respondents rated how bothered they were by each physical symptom on a three-point scale (0 = ‘Not bothered’ to 2 = ‘Bothered a lot’), with total scores ranging from 0 to 28. Prior validation studies in both cultural contexts have confirmed the measure’s reliability and ability to differentiate somatic symptom severity levels.^11,26,27^ Standardised administration protocols were implemented across sites, including quality control checks for incomplete or inconsistent responses, to ensure data comparability.

### Data analysis

The analysis integrated traditional statistical methods with network modelling techniques. Initial descriptive analyses were performed in SPSS 26.0 for Windows 11 (IBM Corp., Armonk, NY, USA; https://www.ibm.com/spss) to characterise demographic and clinical profiles. Group comparisons between Chinese and Rwandan samples were performed using chi-square tests for categorical variables and independent *t*-tests for continuous measures, with adjustments for multiple comparisons where appropriate.

For network analysis, Gaussian Graphical Models (GGMs)^28^ were estimated using R 4.4.0 on Windows 11 (The R Foundation for Statistical Computing, Vienna, Austria; https://www.r-project.org/) to map the relationships among the 23 depressive and somatic symptoms. In the resulting networks, nodes represented individual symptoms (colour-coded by type: orange for depressive, blue for somatic), and edges depicted pairwise regularised partial correlations. Edge thickness corresponded to association strength, with blue edges indicating positive and red edges indicating negative correlations. The Graphical Least Absolute Shrinkage and Selection Operator (GLASSO) algorithm^17^ with model selection guided by the Extended Bayesian Information Criterion (EBIC) was applied to achieve an optimal balance between^28^ network sparsity and connectivity while minimising false positives. Networks were visualised using the Fruchterman-Reingold algorithm via the qgraph package, which positions strongly connected nodes centrally.^29^

Centrality was assessed via expected influence, calculated using the qgraph package, as this metric appropriately accounts for both positive and negative connections in psychological networks. Expected influence scores were z-standardised to facilitate cross-network comparisons.^30^ Bridge symptoms linking depressive and somatic symptom communities were identified using bridge expected influence (BEI) metrics from the networktools package, with the 80th percentile serving as the significance threshold.^16^ Network stability was evaluated through case-dropping subset bootstrapping; correlation stability coefficients (CS-C) above 0.25 (preferably >0.5) were considered acceptable. Edge weight accuracy was assessed via nonparametric bootstrapping with 1000 iterations to generate 95% CIs.

Cross-cultural network comparisons were conducted using the NetworkComparisonTest package.^37^ A permutation test (1000 iterations) evaluated differences in global strength (sum of absolute edge weights), specific edge weights and network structure invariance. All tests were two-tailed with α = 0.05, providing a comprehensive assessment of cultural differences while controlling for multiple comparisons.

## Results

### Demographic and clinical characteristics

The study included 3830 adolescents (China: *n* = 2017; Rwanda: *n* = 1813) with comparable age distributions (China: 15.35 ± 1.56 years; Rwanda: 15.80 ± 1.90 years; *t =* 8.04, *P* < 0.05). Gender distribution differed significantly between groups (χ^2^ = 11.56, *P* < 0.05), with 56.7% male participants in China versus 51.2% in Rwanda. Clinical measures revealed striking cross-cultural differences: depressive symptoms were substantially more prevalent among Rwandan adolescents (54.6 *v*. 29.2%, χ^2^ = 255.34, *P* < 0.001), whereas somatic symptoms were more frequently reported by Chinese participants (71.0 *v*. 64.0%, χ^2^ = 472.37, *P* < 0.001). These patterns were further substantiated by significantly higher total scores on both the PHQ-9 (*t* = 16.69, *P* < 0.001) and the PHQ-15 (*t* = 20.76, *P* < 0.001) in the Rwandan group (Table 1).

**Table 1:** Participants demographics and symptom prevalence Table 1 long description.

Variables	Rwandan adolescents (*N* = 1813)			Chinese adolescents (*N* = 2017)			χ^2^	*t*-value (95% CI)
	*N*	%	*M* ± s.d.	*N*	%	*M* ± s.d.		
Age			15.80 ± 1.90			15.35 ± 1.56		8.04* (0.34, 0.56)
Female	885	48.80		874	43.30		11.56*	
Male	928	51.20		1143	56.70			
Depression symptoms	990	54.60		588	29.20		255.34*	
Somatic symptoms	1160	64.00		1433	71.00		472.37*	
PHQ-9 total score	1813		6.28 ± 5.08	2017		3.57 ± 4.93		16.69* (2.38, 3.02)
PHQ-15 total score	1813		6.59 ± 4.41	2017		3.54 ± 4.65		20.76* (2.76, 3.34)

PHQ-9, Patient Health Questionnaires for depression; PHQ-15, Patient Health Questionnaires for somatic symptoms.

*P < 0.05.

Notably, the proportions of adolescents exceeding established clinical severity thresholds differed significantly between countries. In Rwanda, 45.0% reported minimal to no depressive symptoms (PHQ-9 total score 0**–**4), compared with 70.8% in China, Mild symptoms were reported by 32.4% of Rwandan versus 17.6% of Chinese adolescents, moderate symptoms by 14.2 *v*. 7.1% and moderately severe to severe symptoms by 8.0 *v*. 4.5%, respectively.

A similar pattern emerged for somatic symptoms (PHQ-15). Minimal to no somatic symptoms were reported by 35.0% of Rwandan and 71.0% of Chinese adolescents, mild symptoms by 41.0 *v*. 17.6%, moderate symptoms by 17.7 *v*. 7.9% and severe symptoms by 5.2 *v*. 3.5% (Supplementary Table 1 and Table 2).

### Symptom network structures

**Fig. 1:**
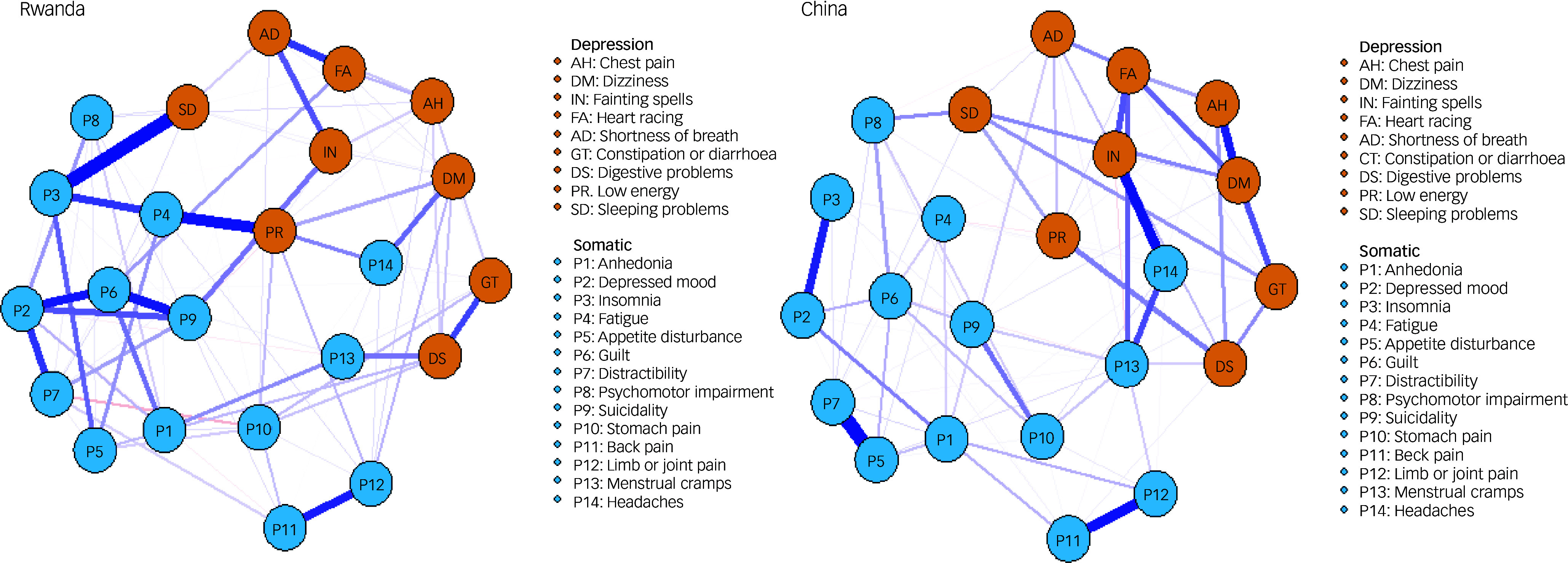
Fig. 1 long description. Network structure of somatic and depression symptoms in Rwandan and Chinese students.

**Fig. 2:**
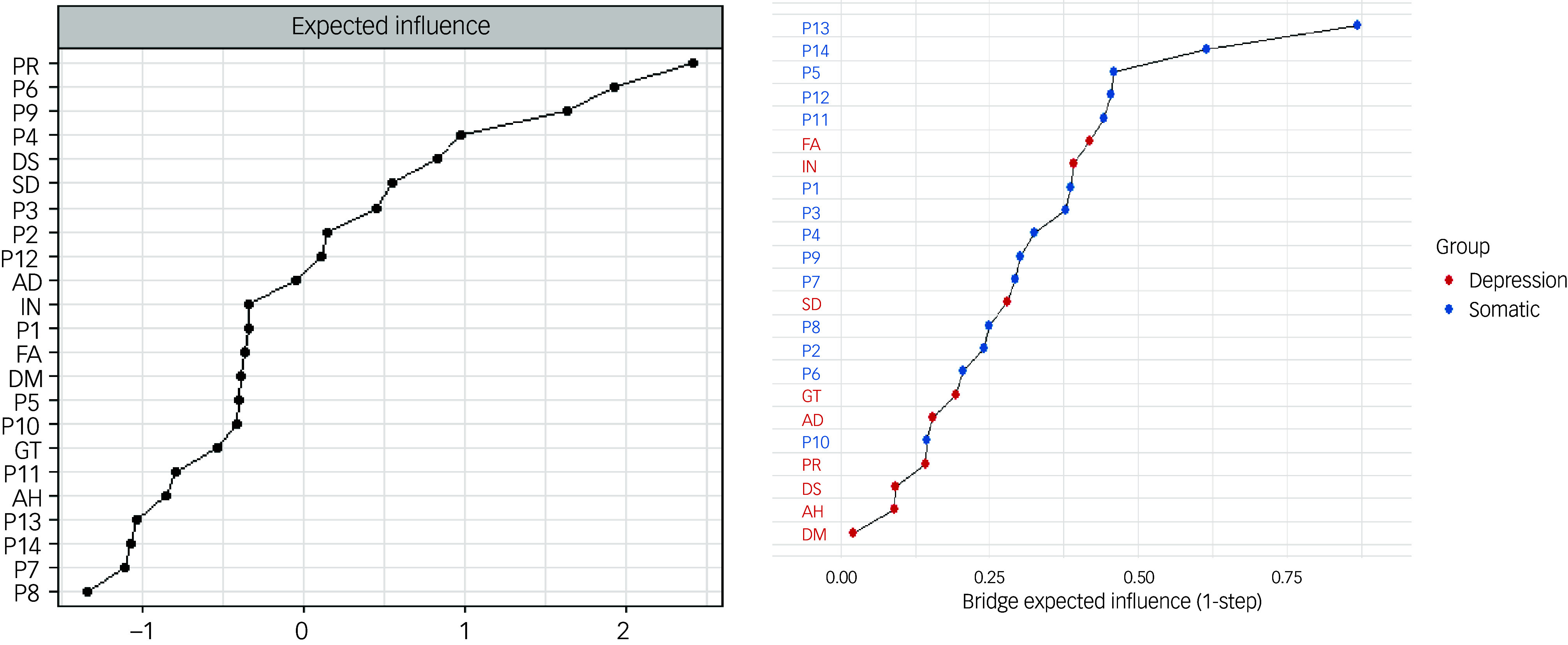
Fig. 2 long description. Expected influence and bridge expected influence of depression and somatic symptoms in Rwandan adolescents. AH, little interest or pleasure in doing things; DM, feeling down, depressed or hopeless; IN, trouble falling or staying asleep, or sleeping too much; FA, feeling tired or having little energy; AD, poor appetite or overeating; GT, feeling bad about yourself – or that you are a failure or have let yourself or your family down; DS, trouble concentrating on things, such as reading the newspaper or watching television; PR, moving or speaking so slowly that other people could have noticed, or being so fidgety or restless that you were moving a lot more than usual; SD, thoughts that you would be better off dead, or thoughts of hurting yourself in some way; P1, stomach pain; P2, back pain; P3, pain in your arms, legs or joints (knees, hips, etc.); P4, menstrual cramps or other problems with your periods (for women only); P5, headaches; P6, chest pain; P7, dizziness; P8, fainting spells; P9, feeling your heart race or pound (palpitations); P10, shortness of breath; P11, constipation, loose stools or diarrhoea; P12, nausea, gas or indigestion; P13, feeling tired or having low energy; P14 trouble sleeping.

**Fig. 3:**
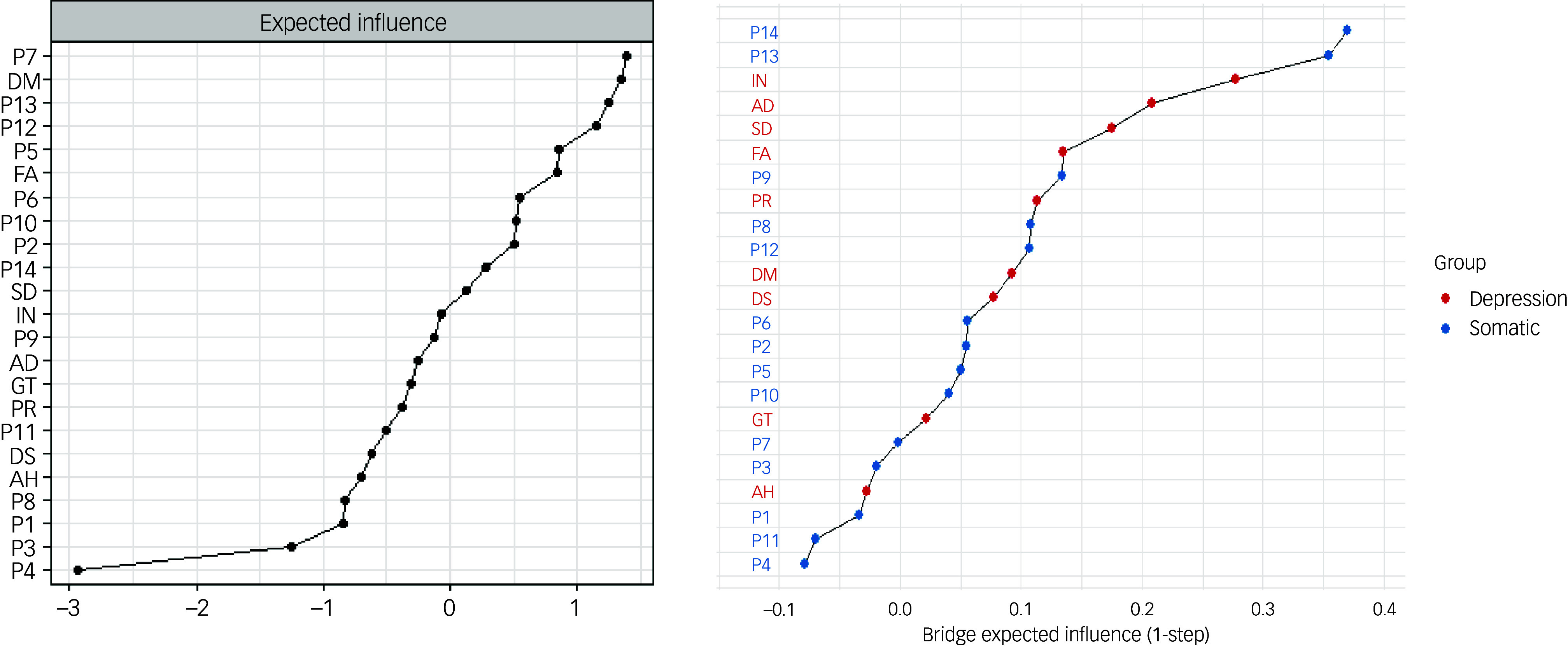
Fig. 3 long description. Expected influence and bridge expected influence of depression and somatic symptoms in Chinese adolescents. AH, little interest or pleasure in doing things; DM, feeling down, depressed or hopeless; IN, trouble falling or staying asleep, or sleeping too much; FA, feeling tired or having little energy; AD, poor appetite or overeating; GT, feeling bad about yourself – or that you are a failure or have let yourself or your family down; DS, trouble concentrating on things, such as reading the newspaper or watching television; PR, moving or speaking so slowly that other people could have noticed, or being so fidgety or restless that you were moving a lot more than usual; SD, thoughts that you would be better off dead, or thoughts of hurting yourself in some way; P1, stomach pain; P2, back pain; P3, pain in your arms, legs or joints (knees, hips, etc.); P4, menstrual cramps or other problems with your periods (for women only); P5, headaches; P6, chest pain; P7, dizziness; P8, fainting spells; P9, feeling your heart race or pound (palpitations); P10, shortness of breath; P11, constipation, loose stools or diarrhoea; P12, nausea, gas or indigestion; P13, feeling tired or having low energy; P14 trouble sleeping.

Network analysis of the 23 depressive and somatic symptoms revealed distinct organisational patterns across cultures. Both networks showed substantial connectivity (154/253 possible edges), but with markedly different central features (Fig. 1). In Rwanda, the strongest connections emerged between sleeping problems and insomnia (edge weight 0.42), suicidal thoughts and guilt (edge weight 0.34) and low energy and fatigue (edge weight 0.33). In contrast, among Chinese adolescents, the strongest edges were observed between dizziness and headaches (edge weight 0.60), constipation/diarrhoea and digestive problems (edge weight 0.51) and insomnia and sleeping problems (edge weight 0.50). Centrality analysis in both samples shows specific symptoms that exert the strongest influence in the network, suggesting they are most strongly connected to other symptoms (Figs 2(a), 3(a)). Symptoms such as psychomotor impairment (expected influence 1.16), guilt (expected influence 1.119) and suicidality (expected influence 0.934) showed as the most influential symptoms in the Rwandan network. In the Chinese network, dizziness (expected influence 1.14), depressed mood (expected influence 1.13) and low energy (expected influence 1.13) showed as the most central symptoms (Table 2).

**Table 2:** Symptom-level analysis of expected influence and bridge expected influence statistics Table 2 long description.

Node	Symptom	Rwanda				China				*P*-value for EI
		*M*	s.d.	EI	BEI	*M*	s.d.	EI	BEI	
PHQ-9 items										
** **AH	Little interest or pleasure in doing things	0.77	0.86	0.609	0.090	0.50	0.75	0.870	−0.028	0.009
** **DM	Feeling down, depressed or hopeless	0.71	0.90	0.753	0.020	0.47	0.74	1.127	0.092	0.009
** **IN	Trouble falling or staying asleep, or sleeping too much	0.75	1.00	0.586	0.391	0.42	0.78	0.909	0.277	0.009
** **FA	Feeling tired or having little energy	0.88	0.90	0.780	0.419	0.46	0.75	1.130	0.134	0.009
** **AD	Poor appetite or overeating	0.70	0.93	0.754	0.155	0.35	0.70	0.893	0.208	0.049
** **GT	Feeling bad about yourself – or that you are a failure or have let yourself or your family down	0.72	1.01	0.714	0.192	0.55	0.86	0.921	0.021	0.009
** **DS	Trouble concentrating on things, such as reading the newspaper or watching television	0.75	0.95	0.913	0.091	0.36	0.73	0.790	0.077	0.069
** **PR	Moving or speaking so slowly that other people could have noticed, or being so fidgety or restless that you were moving a lot more than usual	0.56	0.86	1.164	0.142	0.26	0.62	0.912	0.113	0.009
** **SD	Thoughts that you would be better off dead, or thoughts of hurting yourself in some way	0.43	0.85	0.880	0.280	0.19	0.54	0.809	0.175	0.346
PHQ-15 items										
** **P1	Stomach pain	0.55	0.68	0.810	0.386	0.31	0.55	0.816	−0.034	0.910
** **P2	Back pain	0.53	0.64	0.919	0.241	0.28	0.51	0.950	0.055	0.574
** **P3	Pain in your arms, legs or joints (knees, hips, etc.)	0.58	0.66	0.897	0.377	0.4	0.58	0.747	−0.019	0.009
** **P4	Menstrual cramps or other problems with your periods (for women only)	0.39	0.66	1.027	0.325	0.25	0.50	0.448	−0.078	0.009
** **P5	Headaches	0.82	0.67	0.793	0.458	0.30	0.52	1.066	0.050	0.009
** **P6	Chest pain	0.31	0.66	1.119	0.204	0.14	0.39	0.884	0.055	0.039
** **P7	Dizziness	0.46	0.62	0.742	0.293	0.27	0.51	1.137	−0.001	0.009
** **P8	Fainting spells	0.14	0.40	0.664	0.249	0.06	0.26	0.608	0.107	0.465
** **P9	Feeling your heart race or pound (palpitations)	0.43	0.61	0.934	0.302	0.24	0.49	0.923	0.134	0.920
** **P10	Shortness of breath	0.25	0.51	0.807	0.144	0.19	0.44	0.984	0.040	0.009
** **P11	Constipation, loose stools or diarrhoea	0.35	0.60	0.699	0.442	0.24	0.49	0.841	−0.069	0.049
** **P12	Nausea, gas or indigestion	0.48	0.63	0.821	0.453	0.20	0.46	1.003	0.107	0.009
** **P13	Feeling tired or having low energy	0.85	0.67	0.632	0.869	0.34	0.57	1.145	0.354	0.009
** **P14	Trouble sleeping	0.47	0.64	0.716	0.614	0.33	0.58	0.952	0.369	0.009

EI, expected influence; BEI, bridge expected influence, significance at *P* < 0.05; PHQ-9, Patient Health Questionnaires for depression; PHQ-15, Patient Health Questionnaires for somatic symptoms.

Conversely, differences in the pattern of reporting of depression symptoms were observed across both groups. In the Chinese sample, symptoms including anhedonia, depressed mood, insomnia and feeling tired exhibited significantly higher global influence, while, in contrast, symptoms related to trouble concentrating, psychomotor slowness and suicidal ideation were more frequent in the Rwandan sample.

### Bridge symptoms and network stability

BEI quantified how strongly a symptom connects communities – the symptoms with the highest BEI represent critical pathways linking depressive and somatic domains, potentially facilitating symptom co-activation across clusters. These findings highlighted both shared and culturally distinct patterns (Figs 2(b), 3(b)). Low energy (BEI = 0.87) and sleep problems (BEI = 0.61) were prominent bridges in Rwanda, whereas sleep problems (BEI = 0.37) and low energy (BEI = 0.35) served as primary connectors in China. Headaches also functioned as a notable bridge symptom in Rwanda (BEI = 0.46) (Table 2).

**Fig. 4:**
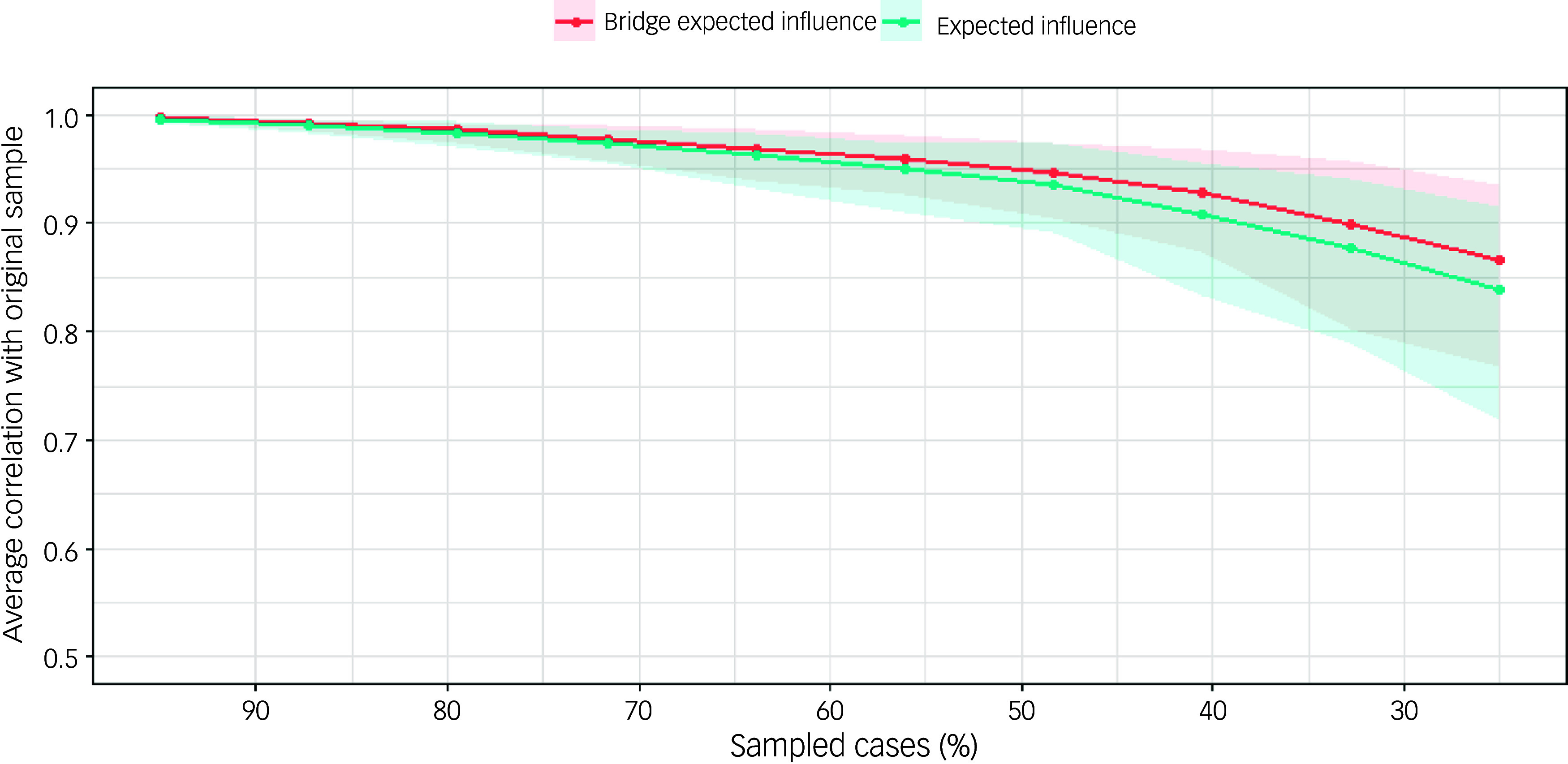
Fig. 4 long description. Flow network of depression-somatic symptoms in the Rwandan group.

**Fig. 5:**
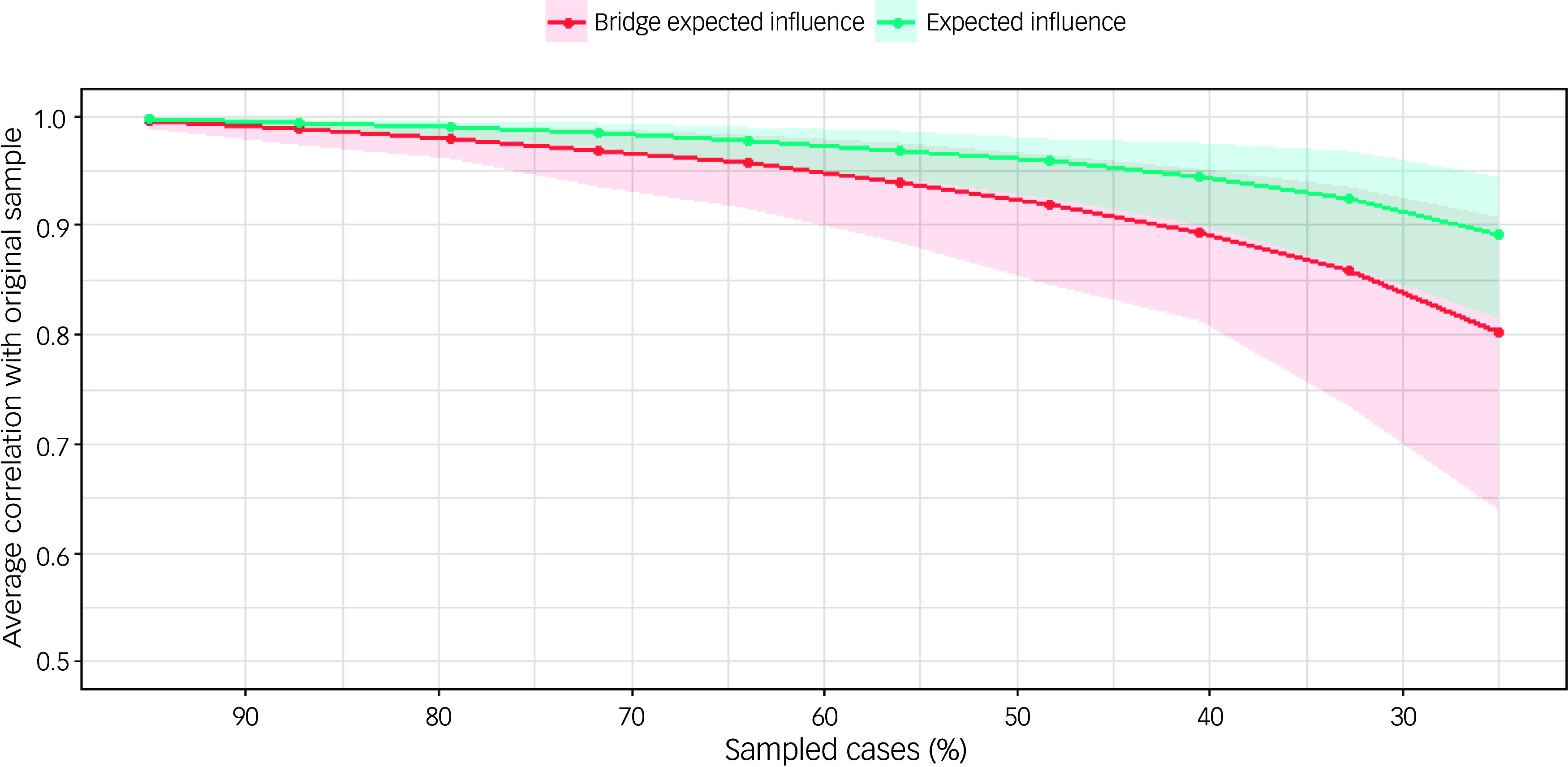
Fig. 5 long description. Flow network of depression-somatic symptoms in the Chinese group.

The stability of centrality indices was examined using case-dropping bootstrap procedures, evaluating how consistently node centrality estimates replicated across subsamples. For the BEI in the Rwandan sample, CS-coefficients remained robust maintained values of 0.92 when retaining 40% of the sample and 0.88 at 30% retention; in the Chinese sample, CS-coefficients demonstrated acceptable stability, maintaining values of 0.89 at 40% sample retention and 0.81 at 30% retention, well above the recommended cut-off of 0.50 for stable interpretation. Expected influence demonstrated similarly strong stability, with CS-coefficients of 0.93 at 40% sample retention and 0.89 at 30% retention in Rwanda and CS-coefficients of 0.94 at 40% retention and 0.91 at 30% retention in Chinese sample, approaching 1.00 at higher retention levels, respectively. These findings indicate that centrality estimates in both samples are reliably estimated and minimally influenced by case selection (Figs 4 and 5).

**Fig. 6:**
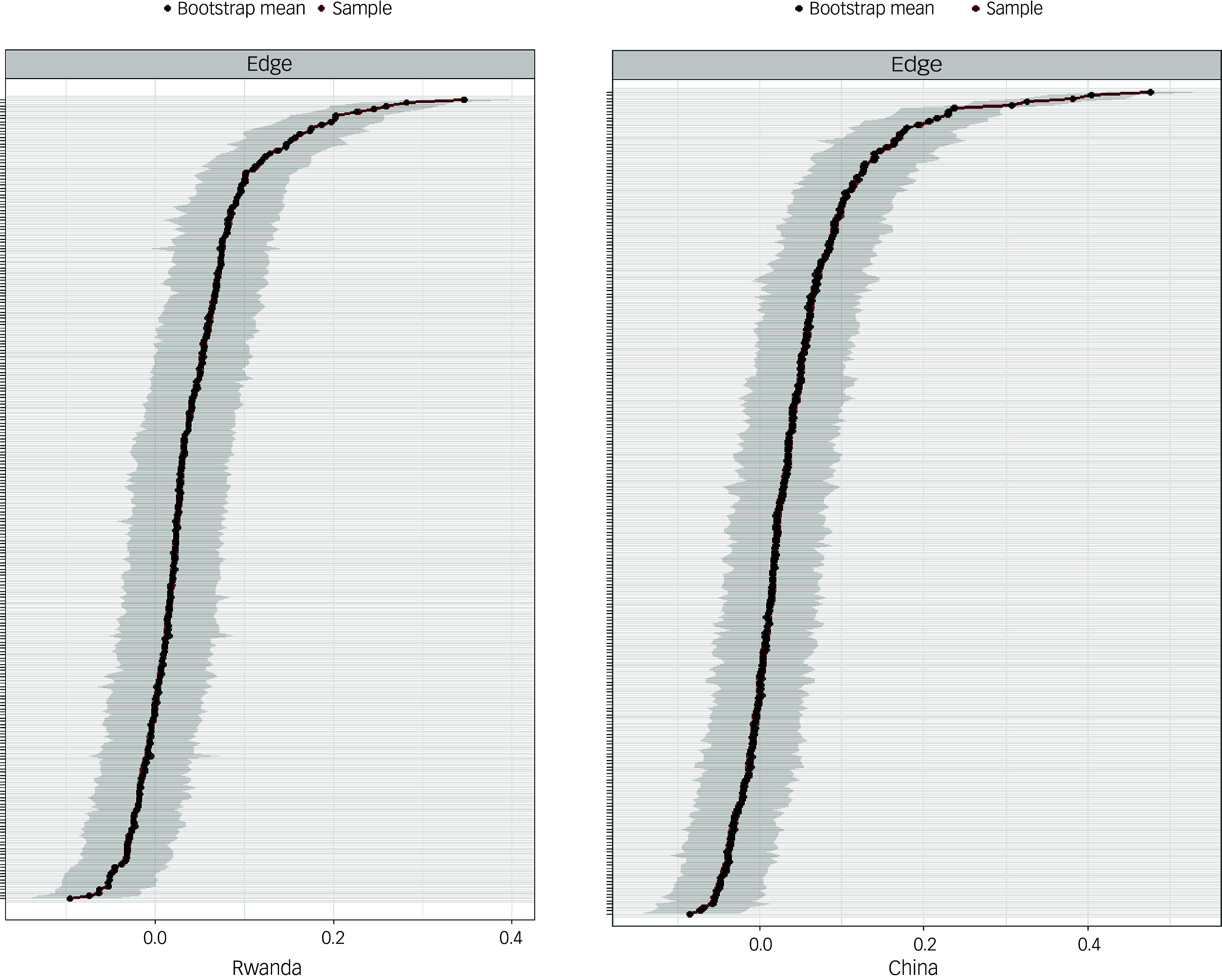
Fig. 6 long description. Edge accuracy plot depicting 95% confidence obtained from 2500 bootstrap samples.

The stability of network edge weights was examined using nonparametric bootstrapping (2500 samples), with 95% CIs derived from the bootstrap distribution. As illustrated in Fig. 6, the edge weights demonstrated acceptable precision, with narrower CIs observed for moderate-to-large edge values (e.g. edges approximating 0.4), indicating greater stability. Conversely, edges with sample estimates near zero exhibited wider CIs, reflecting increased uncertainty. The bootstrap mean estimates closely aligned with the original sample edge weights, suggesting minimal bias in the initial network estimation.

### Cross-cultural network comparisons

Direct comparison revealed statistically significant differences in global network strength (China: 10.44 *v*. Rwanda: 9.44; *S* = 0.99, *p* < 0.05) and overall network structure (*M* = 0.39, *p* < 0.05). Examination of individual edges identified 71 significant differences (*p* < 0.05) among the 253 possible connections. Particularly notable contrasts included stronger connections in the Chinese network for dizziness–headaches (ΔEW = 0.39) and anhedonia–depressed mood (ΔEW = 0.31), whereas psychomotor impairment–suicidal ideation (ΔEW = 0.31) was more prominent in the Rwandan network. Collectively, these findings demonstrate that cultural context fundamentally shapes not only the expression of individual symptoms but also their dynamic interrelationships within adolescent psychopathology networks.

## Discussion

This study presents the first cross-cultural network comparison of somatic-depressive symptoms between Chinese and Rwandan adolescents. Our findings reveal both overlapping and culturally distinct patterns of symptom organisation, demonstrating the utility of network analysis in elucidating culturally mediated expressions of distress and informing targeted mental health interventions.

Consistent with our hypotheses, depression symptoms were markedly more prevalent among Rwandan adolescents (54.6 *v*. 29.2%), whereas somatic symptoms were more frequently reported in the Chinese sample (71.0 *v*. 64.0%). Network analysis further uncovered culturally specific central symptoms: chest pain and psychomotor impairment emerged as dominant nodes in the Rwandan sample, while dizziness and headaches were pivotal in China. Notably, bridge symptoms, particularly low energy and sleep problems, connected somatic and depressive clusters in both groups, albeit with varying influences. These findings aligned with global mental health research highlighting the cultural mediation of symptom expression.^31^

The observed differences likely reflect distinct psychosocial and historical contexts. In Rwanda, the centrality of psychomotor symptoms and the strong connection between guilt and suicidal ideation may be linked by historical trauma exposure and collective guilt narratives.^12,32^ Although participants in this study were born more than a decade after the 1994 genocide, many grew up in families and communities still marked by loss, displacement and disrupted caregiving. Intergenerational transmission of trauma can manifest as heightened physiological arousal, chronic pain and other somatic complaints resembling depressive symptoms.^4^ Such trauma-related somatic expression may be exacerbated by contextual stressors such as poverty, community violence and orphanhood. In conflict-affected African settings, somatic complaints often serve as culturally sanctioned idioms of distress, masking underlying psychological trauma.^15,33^

The prominence of psychomotor impairment may also reflect the biological embedding of chronic stress,^34^ while co-occurring somatic anxiety manifestations (e.g. chest pain, palpitations) illustrate how stigma in low-resource settings may channel psychological distress into bodily expressions.^33^ This contrasts with the symptom architecture observed in Chinese adolescents, where academic stress-related somatic symptoms (dizziness, headaches) prevailed,^35^ consistent with East Asian somatisation tendencies.^6^ Intriguingly, while the connection between depressed mood and anhedonia mirrored Western models, somatic complaints remained prominent, highlighting persistent cultural norms that constrain emotional disclosure despite increasing globalisation of depression concepts.^7^

Our findings both corroborate and extend previous literature. The transdiagnostic role of fatigue and sleep problems aligns with cross-disorder models^36,37^ and prior work in Chinese adults.^8^ However, the centrality of psychomotor symptoms in the Rwandan network diverges from Western patterns, where sadness and anhedonia typically dominate.^31^ Motor symptoms may have been historically underrecognised in African mental health research.^9^ Conversely, China’s dual focus on somatic and cognitive-affective symptoms may reflect an emerging cultural hybridity, where traditional somatisation coexists with Western conceptualisations of depression, especially among urban youth.^7^ Methodologically, this study demonstrates network analysis’ capacity to uncover cultural variations often obscured by conventional assessment approaches.^29^

Contrary to some previous studies suggesting gender differences in somatic reporting, gender did not significantly shape somatic symptom patterns in our sample once depressive symptom severity was considered. This suggests that within the^8,31,34^ cultural contexts examined, somatic symptoms may be driven more strongly by underlying depressive processes and contextual factors than by gender per se. Any observed gender differences may reflect cultural norms surrounding symptom expression rather than substantive differences in psychopathology.

These culturally distinct symptom networks underscore the need for tailored clinical approaches. In Rwanda, clinicians should be attentive to psychomotor impairment and chest pain as potential indicators of underlying distress, particularly among youth. The strong link between suicidal ideation and somatic pain underscores the role of physical symptoms in suicide risk during adolescence.^38^ Conversely, in China, dizziness and headaches may serve as key markers of distress, especially among adolescents facing academic pressure.^35^

Cognitive–behavioural therapy (CBT) protocols that validate somatic complaints while addressing their psychological roots could enhance treatment engagement in both settings.^6^ The transdiagnostic nature of bridge symptoms such as fatigue and sleep disturbances offers promising targets for low-intensity, school- or community-delivered interventions, particularly relevant in Rwanda and China, where mental health stigma remains a barrier to care. These findings do not dictate specific pharmacological choices but highlight the value of culturally adapted symptom-focused strategies, including evidence-based sleep and fatigue management.^15,16^

Several limitations warrant consideration. First, the cross-sectional design precludes causal inferences regarding symptom interactions; longitudinal network analyses are needed to elucidate directional relationships.^29^ Second, reliance on self-report measures may introduce cultural response bias, for instance Rwandan adolescents may underreport psychological symptoms due to stigma, whereas Chinese youth may emphasise somatic complaints.^7^ Future studies would benefit from multi-method assessments, including clinician ratings. Third, differences in sampling procedures (stratified random sampling in China versus cluster sampling in Rwanda) may affect comparability, though both approaches were tailored to local logistical realities and the different data collection methods across sites is to be considered, while as much caution was applied to limit bias; however, the method of data collection may influence symptom reporting and affect endorsement rates. Accordingly, these findings should be interpreted cautiously, as observed cross-cultural differences may partly reflect mode-related reporting biases. Fourth, the restriction to urban settings (Chongqing and Kigali) limits generalisability to rural populations. Finally, while network analysis maps symptom relationships, it does not incorporate external determinants; future research could integrate contextual factors through multilevel modelling.

This study illustrates how cultural context modulates the phenotypic expression of adolescent depression. In Rwanda, symptom networks reflect trauma-associated and stigma-mediated pathways, whereas in China, they manifest as stress-driven somatic-affective loops. These findings highlight the necessity of culturally adapted assessment tools and interventions that prioritise locally salient symptom profiles. Moving forward, integrating network-based insights into clinical training, screening protocols and public health strategies will be essential for advancing equitable and effective adolescent mental health care worldwide.

## Supporting information

10.1192/bjo.2026.12007.sm001 Niwenahisemo et al. supplementary material Niwenahisemo et al. supplementary material

## Data Availability

The data-sets used in the current research study are available from the corresponding authors upon reasonable request.

## Supplementary material

The supplementary material is available online at https://doi.org/10.1192/bjo.2026.12007

## Table 1 Long description

The table compares demographics and symptom prevalence between Rwandan and Chinese adolescents. It includes data on age, gender distribution, and clinical measures such as depression and somatic symptoms. The table has 7 rows and 10 columns. The columns are labeled as follows: Age, Female, Male, Depression symptoms, Somatic symptoms, PHQ-9 total score, and PHQ-15 total score. Each column provides data for both Rwandan and Chinese adolescents, including the number of participants (N), percentage (%), and mean with standard deviation (M ± s.d.). Notable trends include a higher prevalence of depression symptoms among Rwandan adolescents (54.6%) compared to Chinese adolescents (29.2%), and a higher prevalence of somatic symptoms among Chinese adolescents (71.0%) compared to Rwandan adolescents (64.0%). The PHQ-9 and PHQ-15 total scores are also significantly higher in the Rwandan group.

Navigate back to Table 1.

## Table 2 Long description

The table presents a symptom-level analysis of expected influence and bridge expected influence statistics for Rwanda and China. It includes data for various symptoms categorized under PHQ-9 and PHQ-15 items. The table has 15 rows and 12 columns, with columns labeled Node, Symptom, and various statistical measures for Rwanda and China, including M, s.d., BEI, and P-value for EI. Each row lists a specific symptom and its corresponding statistical values for both countries. Notable trends include varying levels of expected influence and bridge expected influence across different symptoms.

Navigate back to Table 2.

## Fig. 1 Long description

The image presents a comparative network structure of somatic and depression symptoms among Rwandan and Chinese students. The network diagrams feature nodes representing different symptoms and edges indicating the relationships between them. The nodes are color-coded: orange for depression symptoms and blue for somatic symptoms. Each node is labeled with abbreviations corresponding to specific symptoms, such as AD for shortness of breath, DM for dizziness, and P1 for anhedonia. The edges vary in thickness, signifying the strength of the relationships between symptoms. The diagrams illustrate how these symptoms interconnect differently in Rwandan and Chinese students, highlighting cultural or contextual variations in symptom networks.

Navigate back to Fig. 1.

## Fig. 2 Long description

A two line graph showing expected influence and bridge expected influence of depression and somatic symptoms in Rwandan adolescents. The x-axis represents bridge expected influence with values ranging from 0.00 to 0.75. The y-axis on the left represents expected influence with values ranging from -1 to 2. The y-axis on the right lists various labels such as P R, P 6, P 9, P 4, D S, S D, P 3, P 2, P 12, A D, I N, P 1, F A, D M, P 5, G T, P 11, A H, P 14, P 13, P 10, P 7, P 8. The graph includes two groups: DEPRESSION and SOMATIC, represented by red and blue dots respectively. The red dots indicate DEPRESSION, and the blue dots indicate SOMATIC. The data points show a trend where the influence increases as the bridge expected influence increases. All values are approximated.

Navigate back to Fig. 2.

## Fig. 3 Long description

The line graph displays two sets of data points representing the expected influence and bridge expected influence of depression and somatic symptoms in Chinese adolescents. The x-axis of the graph ranges from negative three to positive one for expected influence and from negative zero point one to positive zero point four for bridge expected influence. The y-axis lists various symptoms and conditions such as P 7, D M, P 13, P 12, P 5, F A, P 6, P 10, P 2, P 14, S D, I N, P 9, A D, G T, P R, P 11, D S, A H, P 8, P 3, P 1, P 4. The data points are color-coded into two groups: depression and somatic, with depression represented by red dots and somatic by blue dots. The graph shows a clear trend where the influence of symptoms increases from left to right. All values are approximated.

Navigate back to Fig. 3.

## Fig. 4 Long description

A line graph displays the average correlation with the original sample on the y axis and the percentage of sampled cases on the x axis. Two data series are represented: bridge expected influence in red and expected influence in blue. Both lines start at a correlation of one when ninety percent of cases are sampled and gradually decrease as the percentage of sampled cases decreases. The bridge expected influence line shows a steeper decline compared to the expected influence line, indicating a stronger drop in correlation as fewer cases are sampled. The shaded areas around the lines represent confidence intervals, with the blue series having a wider interval as the number of sampled cases decreases. All values are approximated.

Navigate back to Fig. 4.

## Fig. 5 Long description

A line graph displays the average correlation with the original sample on the y-axis and the percentage of sampled cases on the x-axis. Two data series are plotted: bridge expected influence in red and expected influence in blue. The red line representing bridge expected influence starts at a correlation of one and gradually decreases as the percentage of sampled cases reduces, showing a steeper decline compared to the blue line. The blue line representing expected influence also starts at a correlation of one and decreases more gradually. Both lines show a downward trend, indicating a decrease in correlation with fewer sampled cases. The shaded areas around the lines represent the confidence intervals, with the red line having a wider interval as the sampled cases decrease. All values are approximated.

Navigate back to Fig. 5.

## Fig. 6 Long description

The two line graphs compare edge accuracy plots for Rwanda and China. Each graph features a black line representing the bootstrap mean and a red line representing the sample. The x-axis ranges from 0.0 to 0.4, while the y-axis shows the cumulative distribution. The shaded gray area indicates the 95% confidence interval obtained from 2,500 bootstrap samples. Both graphs show a similar upward trend, with the lines closely following each other and the confidence intervals widening as they approach the higher values on the x-axis. All values are approximated.

Navigate back to Fig. 6.
