# Supplementary material for: Cross-cultural comparison of somatic-depressive symptom networks in Chinese and Rwandan adolescents: network analysis study
Source: BJPsych Open. 2026 Jun 26;12(4):e169. doi: 10.1192/bjo.2026.12007 (PMC13312278; doi:10.1192/bjo.2026.12007)
Supplement: Niwenahisemo et al. supplementary material [file S2056472426120079sup001.docx]

RESULTS INTERPRETATION

**proportion of children over symptom thresholds** for each instrument

**Supplementary Table 1 Depression severity (PHQ‑9) by country**

| **PHQ-9** | **Rwanda** | | **χ²** | **df** | **p** | **China** | |
| --- | --- | --- | --- | --- | --- | --- | --- |
|  | **n** | **%** |  |  |  | **n** | **%** |
| None/Minimal | 823 | 45.4 |  |  |  | 1429 | 70.8 |
| Mild | 587 | 32.4 |  |  |  | 355 | 17.6 |
| Moderate | 258 | 14.2 |  |  |  | 143 | 7.1 |
| Moderately sev. | 107 | 5.9 |  |  |  | 60 | 3.0 |
| Sever | 38 | 2.1 |  |  |  | 30 | 1.5 |
| Total | 1813 | 100.0 | 257.22 | 4 | <.001 | 2017 | 100.0 |
| Note. Values are counts and column percentages within country.  PHQ-9 = Patient Health Questionnaire–9, **χ²:** Chi-square, statistical significance at P < 0.001. | | | | | | | |

**Depression severity (PHQ‑9) by country:** Counts and column percentages of participants falling into each PHQ‑9 category (None/Minimal, Mild, Moderate, Moderately severe, Severe) for Rwanda and China; χ²(4, N = 3830) = 257.22, p < .001.

**Supplementary Table 2 Somatic symptom severity by country**

| **PHQ-15** | **Rwanda** | | **χ²** | **df** | **p** | **China** | |
| --- | --- | --- | --- | --- | --- | --- | --- |
|  | **n** | **%** |  |  |  | **n** | **%** |
| None/Minimal | 653 | 36.0 |  |  |  | 1433 | 71.0 |
| Mild | 746 | 41.1 |  |  |  | 354 | 17.6 |
| Moderate | 320 | 17.7 |  |  |  | 159 | 7.9 |
| Severe | 94 | 5.2 |  |  |  | 71 | 3.5 |
| Total | 1813 | 100.0 | 479.17 | 3 | <.001 | 2017 | 100.0 |
| Note. Values are counts and column percentages within country.  PHQ-15 = Patient Health Questionnaire 15, **χ²:** Chi-square, statistical significance at P < 0.001. | | | | | | | |

**Somatic symptom severity (PHQ‑15) by country: Counts and column percentages for None/Minimal, Mild, Moderate, Severe categories; χ²(3, N = 3830) = 479.17, *p* < .001.**

**Supplementary Table 3 Descriptive statistics for somatic symptoms (PHQ‑15) by country and gender**

| **Country** | **Gender** | **n** | **Mean PHQ-15** | **SD** | **95% CI (Lower – upper)** |
| --- | --- | --- | --- | --- | --- |
| Rwanda | Female | 885 | 6.60 | 4.48 | 6.31 - 6.90 |
|  | male | 928 | 6.59 | 4.36 | 6.31 – 6.87 |
| China | female | 874 | 3.35 | 4.52 | 3.05 – 3.65 |
|  | male | 1143 | 3.69 | 7.75 | 3.44 – 3.94 |
| Note: PHQ-15 = Patient Health Questionnaire-15. CI = confidence interval. Means and standard deviations are based on observed (unadjusted) scores. Higher scores indicate greater somatic symptom burden. | | | | | |

Descriptive statistics for PHQ‑15 scores by country and gender: Sample size (n), mean, SD, and 95 % CI for Rwanda (female n = 885, M = 6.60, SD = 4.48; male n = 928, M = 6.59, SD = 4.36) and China (female n = 874, M = 3.35, SD = 4.52; male n = 1 143, M = 3.69, SD = 4.75).

**Supplementary Table 4 ANCOVA results: effect of gender on PHQ‑15 controlling for PHQ‑9, by country**

| **Country** | **Effect** | **df** | **F** | **P** | **Partial η²** |
| --- | --- | --- | --- | --- | --- |
| Rwanda | PHQ-9 scores | 1,1810 | 846.00 | < .001 | .319 |
|  | Gender |  | 0.06 | .812 | .000 |
| China | PHQ-9 scores | 1,2014 | 1723.40 | < .001 | .461 |
|  | Gender |  | 4.74 | .030 | .002 |
| Note:  Note. PHQ‑15 = dependent variable; PHQ‑9 entered as covariate. | | | | | |

**ANCOVA results: effect of gender on PHQ‑15 controlling for PHQ‑9 (separate by country):** Degrees of freedom, F, p, and partial η² for Rwanda (Gender: p = .812, η²p = .000) and China (Gender: p = .030, η²p = .002).

**Table 5 Estimated marginal means of PHQ‑15 by gender and country (controlling for PHQ‑9)**

| **Country** | **Gender** | **Adjusted Mean PHQ-15** | **SE** | **95% CI (Lower – upper)** |  |
| --- | --- | --- | --- | --- | --- |
| Rwanda | Female | 6.57 | 0.12 | 6.33 - 6.81 |  |
|  | male | 6.61 | 0.12 | 6.38– 6.85 |  |
| China | female | 3.35 | 0.12 | 3.13 – 3.58 |  |
|  | male | 3.69 | 0.10 | 3.49 – 3.88 |  |
| Note: PHQ-15 = Patient Health Questionnaire-15; PHQ-9 = Patient Health Questionnaire-9. country-specific sample mean (Rwanda: 6.28; China: 3.57). CI = confidence interval; SE = standard error. | | | | | |

**Estimated marginal means of PHQ‑15 by gender and country (adjusted for PHQ‑9):** When adjusting for depression severity, estimated marginal means for somatic symptoms remained nearly identical for Rwandan women (6.57) and men (6.61). In China, adjusted means were slightly higher for men (3.69) than women (3.35). These results reinforce that depressive symptoms are the primary predictor of somatic burden, with gender playing only a minor role in the Chinese sample and no meaningful role in Rwanda.
